# Detection of non-tuberculous mycobacteria in native wildlife species at conservation risk of Argentina

**DOI:** 10.3389/fvets.2024.1346514

**Published:** 2024-02-14

**Authors:** Soledad Barandiaran, Loreana Ponce, Indiana Piras, Ana Carolina Rosas, Jorge Peña Martinez, María Jimena Marfil

**Affiliations:** ^1^Laboratorio de Tuberculosis, Instituto de Investigaciones en Producción Animal (INPA), Universidad de Buenos Aires-CONICET, Buenos Aires, Argentina; ^2^Cátedra de Enfermedades Infecciosas, Facultad de Ciencias Veterinarias, Universidad de Buenos Aires, Buenos Aires, Argentina; ^3^Fundación Rewilding Argentina, Corrientes, Argentina

**Keywords:** non-tuberculous mycobacteria, native wildlife, conservation, bacteriological diagnosis, molecular identification

## Abstract

**Introduction:**

Non-tuberculous Mycobacteria (NTM) are mainly environmental but can cause opportunistic infections and diseases in humans and animals. Livestock and wild animals can be infected with NTM. In Argentina, there are native wild species facing conservation risks, and they are the focus of protection and reintroduction projects designed to preserve biodiversity in various ecoregions. The aim of this study was to report the presence of NTM in samples collected from four endangered native wild species from nine Argentine provinces, as part of their pre-release health assessment.

**Methods:**

A total of 165 samples from giant anteater, peccary, tapir and pampas deer were obtained, these included either bronchoalveolar or endotracheal lavages, or oropharyngeal, nasopharyngeal or tracheal swabs. Bacteriological culture followed by molecular identification and sequencing were performed.

**Results:**

A total of 27 NTM were detected, including *Mycobacterium avium* subsp. *hominissuis, M. intracellulare, M. terrae, M. gordonense, M. kumamotonense, M. fortuitum, M. saskatchewanense*, and *M. genavense*. Results revealed a 16,36% NTM recovery rate, with the giant anteater showing the highest prevalence among the mammals under study.

**Discussion:**

In Argentina, due to extensive production systems, the interaction between domestic and wild species sharing the same environment is frequent, increasing the exposure of all the species to these NTM. In this way, the transmission of infectious agents from one to another is feasible. Moreover, NTMs might interfere with the diagnosis of bovine tuberculosis and paratuberculosis. These findings emphasize the importance of active health surveillance in conservation programs. It highlights the need to address NTM epidemiology in wildlife and its impact on conservation and public health.

## 1 Introduction

The term “non-tuberculous mycobacteria” (NTM) is the most commonly used expression to refer to species of the genus *Mycobacterium* other than *Mycobacterium tuberculosis* (MTB) and *Mycobacterium leprae* (1). NTM encompasses saprophytic and opportunistic mycobacteria. Within this group, there is the *Mycobacterium avium -intracellulare* complex (MAC), which includes mycobacteria that cause disease in various animal species, while behaving as an opportunistic pathogen in others (2). While mycobacteria within the MTB complex (MTBC) are primarily associated with clinical signs, the role of NTM causing diseases, mainly related with immunocompromised individuals, is increasingly being reported in both humans and animals (3–5).

Wild mammals are susceptible to pathogenic mycobacteria such as *Mycobacterium bovis* (6, 7). When mammalian tuberculosis (mTB) is endemic in the region, *M. bovis* is the most frequently identified mycobacteria in wildlife specimens. Nonetheless, in situations of low or nonexistent prevalence, the identification of NTM becomes more significant (4). Free-ranging wildlife can potentially encounter these environmental mycobacteria within their natural habitat, particularly during foraging and water consumption (4).

In Argentina, mTB is endemic in cattle in almost every region of the country (8, 9). Research efforts directed toward the surveillance of this disease in the local wildlife populations, particularly focusing on invasive alien species, are a recent development. Furthermore, besides the detection of *M. bovis* in both exotic and native species in Argentina, NTMs with relevance in public health and veterinary contexts were identified as well (10–13).

In Argentina, there are programs aimed for the reintroduction and protection of threatened native species in the region (14). Within these programs, there are instances where sample collections are feasible, in activities such as health check-ups prior to the release or translocation of the specimens, and during the capture of individuals for the placement of monitoring collars. Among the species encompassed within such conservation initiatives is the Giant Anteater (*Myrmecophaga tridactyla*). This species is actively engaged in both conservation and reintroduction efforts, as documented in studies by Jiménez-Pérez et al. (15) and Zamboni et al. (14). Furthermore, it holds a threatened status according to the Ministry of Environment and Sustainable Development (16). For this species, poaching is one of the main threats, and many babies are rescued and raised within these conservation programs (14, 15). Another threatened wildlife species involved in conservation programs is the Pampas Deer (*Ozotoceros bezoarticus*), which is considered endangered (16). The reasons for its decline include intense commercial exploitation (for skins and meat), poaching, habitat destruction and alteration, predation by dogs, competition with livestock, and disease transmission by introduced wildlife species (17). Similarly, the tapir (*Tapirus terrestris*) benefits from a Conservation Action Program (18) and holds a threatened status (16). Uncontrolled sport hunting and the reduction of forested areas are among the leading causes of its disappearance. Lastly, the collared peccary (*Pecari tajacu*) is involved in reintroduction programs (14, 19) and is also classified as a threatened species, with its primary threat being hunting (17).

Few reports are available where epidemiological surveillance of mycobacteriosis is conducted on samples taken from alive native wildlife, especially those with conservation risk, as in this study. Usually, sampling is carried out on tissue from deceased animals within surveillance programs, roadkill, or as part of population control efforts (4, 20). When sampling alive animals, especial conditions are needed, as the collection must be fast as the animals are anesthetized and also, sampling is not invasive most of the times.

Investigating the health condition of native wildlife in a certain region would help protect biodiversity in that ecoregion. This research aims to evidence the presence of NTM in free-ranging native wild animals with different degrees of conservation concern in Argentina.

## 2 Materials and methods

**Figure 1:**
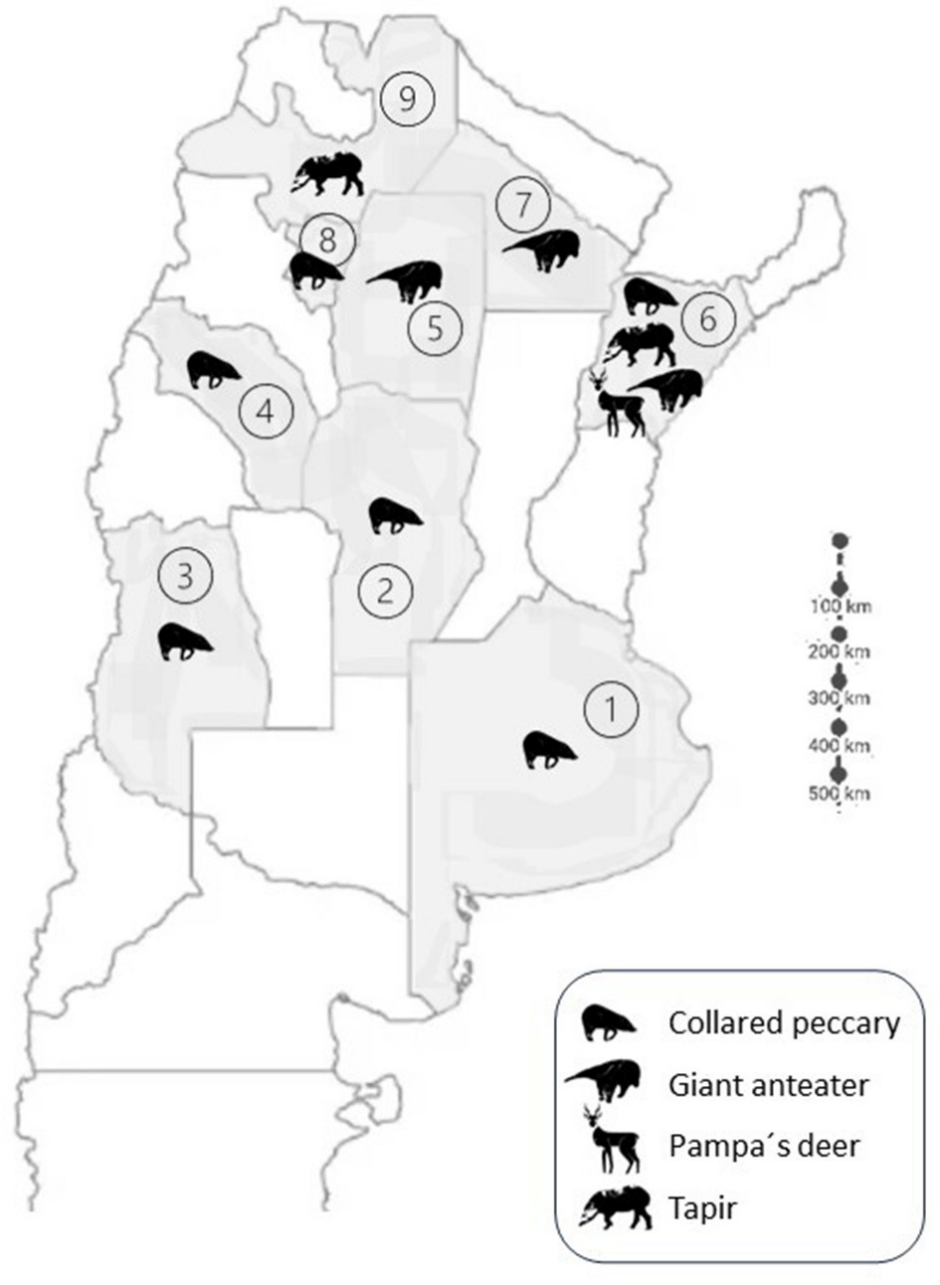
Partial Argentinean map showing the provinces where the native wildlife species are present. The different species are represented with the animal silhouette in each province. References: Provinces: 1. Buenos Aires; 2. Córdoba; 3. Mendoza; 4. La Rioja; 5. Santiago del Estero; 6. Corrientes; 7. Chaco; 8. Tucumán; 9. Salta.

Between the years 2016 to 2021, the Laboratory of Tuberculosis Diagnosis of the Infectious Diseases Department in the Faculty of Veterinary Science at the University of Buenos Aires received 165 (each corresponding to only one animal: 104 Collared peccary, 31 Pampa's deer, 19 Tapir and 11 Giant anteaters) samples from anesthetized native mammals from 9 provinces of Argentina, including Buenos Aires, Chaco, Córdoba, Corrientes, La Rioja, Mendoza, Salta, Santiago del Estero y Tucumán as part of the health checks within conservation programs. The sampled animal species present in each province is shown in Figure 1. The anesthetic protocol adhered to the standard procedures of each institution during these procedures and was carried out by the institution's responsible veterinarian group, following guidelines that ensure animal's welfare (21). The received samples included bronchoalveolar lavages, endotracheal lavages, oropharyngeal swabs, nasopharyngeal swabs, and tracheal swabs. The conservation categories of each native wildlife species were determined in accordance with Resolution 316/2021 from the Ministry of Environment and Sustainable Development. The samples were kept frozen at−20°C until shipment and processing in the Laboratory of Tuberculosis Diagnosis (FCV-UBA).

### 2.1 Bacteriological culture

The bacteriological culture of the submitted samples was performed using the Löwenstein-Jensen medium. The Petroff decontamination technique was applied beforehand, treating rhe sample with NaOH (4%) and the sediment obtained is hen neutralized with HCl and placed in the sterile medium, as specified bu Jorge et al. (22). Cultures were incubated at 37°C for up to 12 weeks, and those with no bacterial growth were discarded as negative (22). Colonies compatible with mycobacteria growth were stained using the Ziehl-Neelsen technique for acid-fast bacilli (AFB) observation.

### 2.2 Molecular diagnosis

For the detection of the genre *Mycobacterium* in the isolates, DNA from the bacteriological cultures were obtained by thermal lysis. For this purpose, a colony was taken with a sterile 1 μL loop and suspended in 300 μL of sterile pyrogen-free distilled water in 1.5 mL RNase-free microtubes. Subsequently, the microtubes were subjected to 95°C for 40 min and centrifuged for 10 min at 12,000 rpm. DNA was kept frozen at −20°C until processing. This DNA was subjected to PCR amplification of the heat shock protein 65 kD (*hsp*65) using the primers TB11 (ACCAACGATGGTGTGTCCAT) and TB12 (CTTGTCGAACCGCATACCCT) and the cycling described by Telenti et al. (23). All PCR products were included in a 2% agarose gel stained with Ethidium bromide (0.5 μL/mL) and observed under a UV light. Those isolates shielding a band at 440 bp were considered positive. These isolates were further studied by sequencing and analyses. Some isolates from different species were selected for sequencing due to economic considerations, aiming to represent each species of wild animal and considering the quality of the band observed in the *hsp*65 PCR. The selected PCR products were purified using one of the following purification kits “Illustra DNA and Gel Band Purification Kit” (GE Healthcare, UK) or “GFX™ PCR DNA and Gel Band Purification Kit” (Cytiva, USA) following manufacturer's specifications. Purified products' quality was confirmed by quantification in a spectrophotometer at a wavelength of 260 nm (Nanodrop 2000, Thermo Scientific™, Thermo Fisher Scientific, USA).

For the detection of the *Mycobacterium avium* complex (MAC) members, PCR amplification of the insertion sequence *1245* was performed. Differentiation between species within the complex was achieved through amplification of insertion sequence *901*. For IS*1245*, amplification was performed using primers P1 (GCCGCCGAAACGATCTAC) and P2 (AGGTGGCGTCGAGGAAGAC) and the cycling described by Guerrero et al. (24). A band weighing 427 bp was considered a positive sample for MAC. All MAC-positive isolates were further studied for subspecies detection amplifying the insertion sequence *901*, using primers P1 (GGATTGCTAACCACGTGGTG) and P2 (GCGAGTTGCTTGATGAGCG) and the cycling described by Moravkova et al. (25). When a PCR product of 577 bp was observed, *Mycobacterium avium* subsp. *avium* was identified, while when no bands were observed *Mycobacterium avium* subsp. *hominissuis* was identified.

### 2.3 Sequencing and analyses

Sequencing was carried out in the Genomics Unit of the Institute of Biotechnology, in the Institute of Agrobiotechnology and Molecular Biology (IABIMO INTA-CONICET) for the Giant anteater, Tapir and some Pampas deer, and for the collared peccary by Macrogen sequencing service (Korea). The institution that provided sequencing changed during the years these animals were sampled, but both institutions performed the same sequencing, using a 16-capillary sequencer ABI3130xl (Applied Biosystems, Thermo Fisher Scientific, USA), using “Big Dye Terminator v3.1” (Cycle Sequencing Kit). The sequences that shielded good quality were compared to those in the National Center of Biotechnology Information using the Basic Local Alignment Search Tool (BLAST). Identification was based on similarities between our isolates and those in the database, identifying the best match considering the percentage of coverage and identity.

## 3 Results

A total of 27 NTM were detected from the 165 (16,36%) investigated samples. The frequency and identity of the *Mycobacterium* and the frequency in each animal species can be observed in Table 1. Twenty-seven *hsp*65 positive isolates were subjected to IS*1245* PCR and in 4 samples MAC was detected, all of them were IS*901* negative, being identified as *M. avium* subsp. *hominissuis*. From the remaining IS*1245* negative samples, 20 samples were sent for sequencing. Not all the *Mycobacterium* revealed a clear species identity and coverage when compared to those in the BLAST online database, being identified with the same percentage of identity and coverage as more than two species or only being identified as “*Mycobacterium* spp.”. Those with no clear identification were kept as “*Mycobacterium* spp.” for this work. In regards to the giant anteater, the species identified were: *M. avium* subsp. *hominissuis* (1/5), *M. terrae* (1/5), *M. gordonae* (1/5) and the rest were identified as *Mycobacterium*. spp (2/5). In regards to the tapir, *M. genavense* (1/6), *M. saskatchewanense* (1/6), *M. intracellulare* (1/6) and three other *Mycobacterium* spp were identified. Regarding the collared peccary, *M. avium* subsp. *hominissuis* (2/10), *M. terrae* (1/10), *M. kumamotonense* (1/10), *M. fortuitum* (1/10) and five other *Mycobacterium* spp. (5/10) were identified. Lastly, regarding pampas deer, *M. avium* subsp. *hominissuis* (1/4), *M. intracellulare* (1/4) and two other *Mycobacterium* spp. (2/4) were detected. The most frequent *Mycobacterium* species detected were *M. avium* subsp. *hominissuis* and *M. terrae*. The animal species with the highest Mycobacteria detection was the giant anteater.

**Table 1:** Frequency of the identified Mycobacteria in the native wildlife species.

**Province**	**Animal species**	**Sample**	**Isolated**	***n***
La Rioja	Collared peccary	Tracheal swab	*M. terrae*	2
			*Mycobacterium* spp.	3
Mendoza	Collared peccary	Tracheal swab	*M. avium* subsp. *hominissuis*	1
			*Mycobacterium* spp.	1
Tucumán	Collared peccary	Tracheal swab	*M. avium* subsp. *hominissuis*	1
			*M. kumamotonense*	1
			*M. fortuitum*	1
			*Mycobacterium* spp.	1
Corrientes	Giant anteater	Oropharyngeal swabs	*M. avium* subsp. *hominissuis*	1
			*Mycobacterium* spp.	1
	Tapir	Bronchoalveolar lavages	*M. genavense*	1
		Oropharyngeal swabs	*Mycobacterium* spp.	1
			*M. saskatchewanense*	1
	Pampa's deer	Lavages endotracheal	*M. avium* subsp. *hominissuis*	1
			*M. intracellulare*	1
			*Mycobacterium* spp.	2
Salta	Tapir	Bronchoalveolar lavages	*Mycobacterium* spp.	1
		Oropharyngeal swabs	*M. intracellulare*	1
			*Mycobacterium* spp.	1
Córdoba	Collared peccary	Tracheal swab	*Mycobacterium* spp.	1
Santiago del Estero	Giant anteater	Oropharyngeal swabs	*M. terrae*	1
			*Mycobacterium* spp.	1
Chaco	Giant anteater	Oropharyngeal swabs	*M. gordonae*	1
Total				27

## 4 Discussion

Our study reportsa high NTM recovery rate (16,36%; 27/166) in samples from native wildlife species from different regions of Argentina. None of the sampled animals exhibited clinical signs associated with chronic disease prior to sample collection. Furthermore, from the time of sample collection until the writing of this manuscript, no health data were obtained from these animals. Therefore, it remains unknown whether they developed clinical signs or lesions at any point in their lives.

The animal species with the highest presence of NTM was the giant anteater (45,5%; 5/11). This high recovery of mycobacteria in the oral mucosa of this species might be associated with the way this animal feeds and the distinctive characteristics of its tongue, which is softer, wetter, and rougher, allowing it to adhere to objects before ingestion (26). The presence of NTM such as *Mycobacterium fortuitum* in this species has been reported previously (27). We did not find any literature describing MTBC infection in this species. This could be due to various reasons, such as limited research on the species, potential resistance of the species to pathogenic mycobacteria, or the possibility that environmental mycobacteria colonize the oropharyngeal mucosa, and potentially regulate or interfere with the colonization of pathogenic mycobacteria, directing the mucosal immune response as has been suggested by other authors in human medicine (28–30). More research is required to corroborate this statement for this species.

Tapirs showed a 32% (6/19) prevalence of NTM. This species is reported to be highly susceptible to both *M. bovis* and *M. tuberculosis* (31–34). Given the tapir's high susceptibility to *M. bovis* and the fact that they were moved from regions where mTB is endemic (7), a comparative intradermal tuberculin test (SICCT) was additionally performed, using both Purified Protein Derivatives (PPD) (bovine and avian) applied on the edge of the ear. This test was negative for both PPDs in all the cases. These results imply a higher sensitivity in detecting NTM from pharyngeal swabs and bronchoalveolar lavage samples through bacteriological culture compared to the SICCT. With these results, we support the assertion that the comparative SICCT, although it is recommended to detect *M. bovis*, is definitely an inadequate test for the detection of NTM infections in tapirs, as observed by Marcordes et al. (32) in his study.

In the collared peccary, an incidence of 11,5% (12/104) of NTM was detected. In a study conducted in the same region, Brazil, on 330 samples of peccary lymph nodes, a 3% NTM was detected (35). There are reports describing susceptibility to *M. bovis* infection in this species, and they have been suggested as a possible reservoir for free-ranging animals in some areas of Brazil (35, 36).

The pampas deer is a threatened species in our territory and declines in its wild populations are reported annually (37). In this study, 13% (4/31) of NTM were identified. The *Cervidae* family is highly susceptible to mTB, both in free-ranging and captive animals (38). Several species of deer have been officially recognized as reservoirs of mTB in several countries across the globe (39–42). There are also reports that demonstrate the presence of NTM in this family (43).

Although the presence of NTM might interfere with the routine mTB diagnostic tests, this is more frequent in areas where TB prevalence in cattle is low. Other authors from Spain had reported *M. avium* subsp. *avium* and *hominissuis* and *M. nonchromogenicum* as the most common NTM identified in TST-reactors cattle (4). Another author reports that sensitization with *M. nonchromogenicum, M. intracellulare, M. avium* subsp. *paratuberculosis* and *M. avium* subsp. *hominissuis*, within others, could make animals react to the SITT (44). In Argentina, a study conducted by Oriani et al. (12), in which cattle were inoculated with NTM isolated from soils and wetlands, this NTM included *M. kansasii, M. nonchromogenicum, M. gordonae, M. arupense, M. phlei, M. fortuitum* and *M. peregrinum*, and showed that they may cause unspecific reactions, but that these reactions are not maintained over time (12).

As a limitation of this study, we mention the type of samples collected and analyzed for diagnosis. Given that the animals under study were alive, obtaining tissue samples was not feasible. The samples analyzed were restricted to those that could be collected during the examination of the oral and respiratory cavities, knowing that in the literature, the most representative samples for NTM and *M. bovis* detection are head, mediastinal, and mesenteric lymph nodes from deceased animals (37, 39).

The 16S ribosomal RNA and ***hsp***65 sequencing are both effective for identifying bacteria, particularly *Mycobacterium* species. ***Hsp***65 sequencing yields comparable results to the widely used 16S ribosomal RNA, as reported by various studies, including one conducted in our laboratory on NTM) in animal samples (10). Other authors also confirm that the use of either 16S ribosomal ARN or ***hsp***65 would allow the identification of *Mycobacterium* spp. And, in many cases, to the species level (45). Moreover, a combination of more than one sequence could strengthen the identification of the species, using a combination of at least three different sequences. Also, the use of multilocus sequence typing would improve the identification of mycobacterial species (46, 47). Additionally, poor quality or incorrectly identified sequences could limit the identification when compared against the BLAST database (4, 48).

The studied animals came from different provinces to Corrientes province, where they were relocated. These provinces are located far from each other and have different soil and climate conditions, but no significant clustering of NTM species in each region was observed. Among the Mycobacteria detected in this study, ubiquitous environmental bacteria were isolated, such as *M. avium, M. gordonae, M. terrae, M. fortuitum, M. kumamotonense*. These Mycobacteria can be isolated from soil, water and occasionally have been associated with disease in animals and humans (3, 49–55). *M. avium* has been isolated from several wild species (4) and MAH has already been reported in wild and domestic animals in Argentina (56). *M.intrcellulare* has been reported causing disease in a capybara subjected to stressful conditions and causing lesions similar to other pathogenic Mycobacteria (5). *M. gordonae* and *M. terrae* have been isolated from sputum samples in human patients with respiratory disease (3, 57), but there are no reports of these agents' causing disease in domestic and wild animals. *M. kumamotonense* has been documented in immunocompetent individuals with latent tuberculosis and patients with multiple spiculated pulmonary nodules without respiratory symptoms (58, 59). Other NTM such as *M. saskatchewanense* and *M. genavense* are found in clinical samples from humans in North America and Europe, acting as opportunistic pathogens in immunocompromised patients (60, 61). *M. genavense* has been reported in various wild and domestic animals, including birds, rabbits, cats, ferrets, snakes, and dogs (62–64). According to most authors, transmission to humans occurs through oral ingestion from contaminated water or close contact with infected animals. Additionally, a study in the Serengeti ecosystem, focusing on NTM, found *M. fortuitum* to be a prevalent species. This *Mycobacterium* was identified in cattle tissues and in the sputum of humans showing clinical signs suggestive of tuberculosis (20). NTM detected in this study have been previously reported in soil, water and cattle and wildlife in Argentina (12, 13, 65).

Emphasizing the importance of infections caused by NTM in human medicine is crucial. The prevalence of NTM in humans is increasing, and there is a belief that in certain industrialized countries, it might exceed the incidence of tuberculosis caused by MTBC (66). Additionally, it is known that NTM has developed resistance to most conventional antibiotics, making treatments ineffective and underscoring their profound impact (67). Although the interaction between human and wildlife is occasional in developed countries, in developing countries the human-wildlife interface is becoming increasingly frequent. Therefore, it is important to understand the distribution of mycobacteria in wildlife from different regions, since the information is very scarce. The active surveillance of wildlife reflects what is happening in the environment, which is the primary source of infection for both humans and coexisting animals (68).

The transmission of NTM between domestic and wild species can occur through direct contact but is largely mediated by shared environments (68, 69). The presence of NTM in free-ranging animals that share their environment with livestock highlights the need to differentiate mycobacteria species, because of the potential interference in diagnostic tests, to control mTB (4, 68, 70). In Argentina, there is a particular scenario where extensive livestock farming is the most frequent strategy, allowing domestic and wild animals to interact in the same environment, increasing the likelihood of disease transmission between them, compared to more confined and intensive farming scenarios (56, 69). The adverse consequences associated with the introduction of livestock into habitats occupied by native fauna have been extensively documented, primarily due to the spread of infectious and parasitic diseases (71–78). The same scenario was observed in tapir (79), giant anteaters (80, 81), and peccaries (35). In regards to the peccaries, efficient transmission is also described between different wild species, such as the invasive exotic wild boar and the vulnerable native peccary (69–82).

Our study provides valuable insights into the presence and diversity of NTM in Argentina's native wildlife. This emphasizes the importance of active surveillance, highlighting potential risks to native species and advocating for conservation strategies to mitigate infectious diseases' impacts in shared environments.

## Data availability statement

The datasets presented in this study can be found in online repositories. The names of the repository/repositories and accession number(s) can be found below: https://www.ncbi.nlm.nih.gov/genbank/, MW043443; https://www.ncbi.nlm.nih.gov/genbank/, MW043444.

## Ethics statement

Ethical approval was not required for the studies involving animals in accordance with the local legislation and institutional requirements because animals are wildlife in the context of Conservation and Translocation Programs. Fundación Rewilding Argentina has permits for all the activities they control. The Laboratory of Tuberculosis Diagnosis received the samples and no Ethics Committee is required. Written informed consent was not obtained from the owners for the participation of their animals in this study because this animals have no owners or where translocated from zoos or rescue facilities. Permits are not required as quarantine is mandatory and this tests are in the context of health check-ups.

## Author contributions

SB: Conceptualization, Formal analysis, Funding acquisition, Investigation, Project administration, Supervision, Writing—original draft, Writing—review & editing. LP: Methodology, Writing—original draft, Writing—review & editing. IP: Methodology, Writing—original draft, Writing—review & editing. AR: Methodology, Visualization, Writing—review & editing. JP: Methodology, Visualization, Writing—review & editing. MM: Conceptualization, Formal analysis, Investigation, Methodology, Writing—original draft, Writing—review & editing.
